# Roles of Phosphate in Skeleton

**DOI:** 10.3389/fendo.2019.00180

**Published:** 2019-03-26

**Authors:** Toshimi Michigami, Keiichi Ozono

**Affiliations:** ^1^Department of Bone and Mineral Research, Research Institute, Osaka Women's and Children's Hospital, Osaka Prefectural Hospital Organization, Izumi, Japan; ^2^Department of Pediatrics, Osaka University Graduate School of Medicine, Suita, Japan

**Keywords:** phosphate, mineralization, matrix vesicle, osteoblast/osteocyte, rickets, sensing

## Abstract

Phosphate is essential for skeletal mineralization, and its chronic deficiency leads to rickets and osteomalacia. Skeletal mineralization starts in matrix vesicles (MVs) derived from the plasma membrane of osteoblasts and chondrocytes. MVs contain high activity of tissue non-specific alkaline phosphatase (TNSALP), which hydrolyzes phosphoric esters such as pyrophosphates (PPi) to produce inorganic orthophosphates (Pi). Extracellular Pi in the skeleton is taken up by MVs through type III sodium/phosphate (Na^+^/Pi) cotransporters and forms hydroxyapatite. In addition to its roles in MV-mediated skeletal mineralization, accumulating evidence has revealed that extracellular Pi evokes signal transduction and regulates cellular function. Pi induces apoptosis of hypertrophic chondrocytes, which is a critical step for endochondral ossification. Extracellular Pi also regulates the expression of various genes including those related to proliferation, differentiation, and mineralization. *In vitro* cell studies have demonstrated that an elevation in extracellular Pi level leads to the activation of fibroblast growth factor receptor (FGFR), Raf/MEK (mitogen-activated protein kinase/ERK kinase)/ERK (extracellular signal-regulated kinase) pathway, where the type III Na^+^/Pi cotransporter PiT-1 may be involved. Responsiveness of skeletal cells to extracellular Pi suggests their ability to sense and adapt to an alteration in Pi availability in their environment. Involvement of FGFR in the Pi-evoked signal transduction is interesting because enhanced FGFR signaling in osteoblasts/osteocytes might be responsible for the overproduction of FGF23, a key molecule in phosphate homeostasis, in a mouse model for human X-linked hypophosphatemic rickets (XLH). Impaired Pi sensing may be a pathogenesis of XLH, which needs to be clarified in future.

## Introduction

Phosphorus mediates almost all biological processes including composition of cell membrane, maintenance and inheritance of genetic materials as nucleic acids, energy metabolism, and regulation of proteins by phosphorylation/dephosphorylation, as well as skeletal mineralization in vertebrates (1). In human adult body, about 90% of total phosphorus is stored in bone as hydroxyapatite (calcium-phosphate) crystals (2). Most of the remaining phosphorus is distributed in soft tissues, and phosphate is predominantly an intracellular ion. Less than 1% of phosphorus exists in extracellular fluid (2), and phosphorus in serum mostly occurs as inorganic phosphate (Pi) such as ${\text{HPO}}_{4}^{2-}$ and H_2_${\text{PO}}_{4}^{-}$, and the former corresponds to 80% at physiological pH (3). Although serum Pi level is influenced by age, diet, and pH (1), its abnormality may lead to undesirable consequences.

Since phosphate is an essential constituent of hydroxyapatite, its chronic deficiency or wasting leads to impaired skeletal mineralization characteristic to rickets, and osteomalacia. In addition to its role in hydroxyapatite formation, Pi also induces apoptosis of hypertrophic chondrocytes as a direct effect on cells (4–8). Moreover, extracellular Pi triggers signal transduction to regulate gene expression (9–13).

In this article, after overviewing the mechanism of phosphate homeostasis and hyperphosphatemic and hypophosphatemic disorders, we will describe the versatile roles of phosphate in the skeleton.

## Phosphate Homeostasis in Mammals

### Phosphate Balance

In mammals, phosphate homeostasis as a total body is maintained by influx and efflux via the intestines, kidneys, bone, and soft tissue. In growing children, the phosphate balance should be positive to allow accrual of phosphate in the skeletons and soft tissues. To meet the needs of Pi for the growing skeleton and soft tissues, serum Pi levels are maintained higher in children than in adults (14). This is contrast to serum calcium, which is kept constant. Since intracellular concentrations of Pi are higher than its extracellular concentrations, Pi is taken up into cells by active transport through sodium/phosphate (Na^+^/Pi) co-transporters (15). Although the mechanism for the age-dependent change in serum Pi levels remains unclear, we speculate that the sensing of Pi and the set point of optimal Pi levels might be influenced by skeletal growth and maturation.

### Intestinal Absorption of Phosphate

Dietary phosphate is absorbed in the small intestine by a passive, paracellular diffusion, and an active, transcellular transport of Pi (16). The latter is mediated by type IIb Na^+^/Pi co-transporter (NaPi-IIb) localized in the apical membrane of the small intestine epithelial cells. The intestinal expression of NaPi-IIb is increased by low dietary intake of phosphate and 1,25-dihydroxyvitamin D (1,25(OH)_2_D), an active metabolite of vitamin D (17). Dietary deficiency of calcium is common because calcium content is relatively low in most foods (18). On the other hand, dietary deficiency of phosphate is rare, because all foods are derived from cells consisting of high amount of phosphate.

### Renal Reabsorption of Phosphate

Pi is excreted from the kidneys. After filtered by the glomeruli, majority of Pi is reabsorbed by type IIa and IIc Na^+^/Pi co-transporters (NaPi-IIa and NaPi-IIc) predominantly expressed in proximal tubules (19). Loss-of function mutations in the *SLC34A3* gene encoding NaPi-IIc cause hereditary hypophosphatemic rickets with hypercalciuria, which is characterized by hypophosphatemia due to an increased urinary loss of phosphate (20). The Pi-transport activity of NaPi-IIa and NaPi-IIc in the proximal tubules is determined by their protein amounts in the brush border membrane (BBM).

### Parathyroid Hormone (PTH) and Fibroblast Growth Factor 23 (FGF23)

The amount of NaPi-IIa and NaPi-IIc in the BBM is regulated both transcriptionally and post-transcriptionally through protein synthesis, degradation and subcellular localization, and is regulated by several hormones such as parathyroid hormone (PTH) (21, 22) and fibroblast growth factor 23 (FGF23) (23–26) as well as dietary phosphate intake (27). PTH causes a rapid decrease of NaPi-IIa protein on the BBM (21, 22). The amount of NaPi-IIc on the BBM is also reduced by PTH, although it takes longer time (28, 29). FGF23 is mainly produced by the osteoblasts and osteocytes and exerts its effects on distant organs such as the kidneys in an endocrine manner (30). FGF23 requires αKlotho as a co-receptor to evoke signals through FGF receptors (FGFRs) at physiological concentrations (31, 32). In the kidneys, FGF23 decreases the expression of NaPi-IIa and NaPi-IIc to increase the renal Pi excretion (23–26). In addition, FGF23 reduces the production of 1,25(OH)_2_D by suppressing the expression of vitamin D 1α-hydroxylase and inducing that of 24-hydroxylase, which leads to the decreased Pi absorption in the intestine (24, 33). Considering that FGF23 is unable to normalize serum Pi levels in hyperparathyroidism and that inactivating mutations of FGF23 cause hyperphosphatemia despite the presence of PTH (34), both hormones appear to be necessary to maintain Pi homeostasis.

### Type III Na^+^/Pi Co-transporters

Type III Na^+^/Pi co-transporters include PiT-1 and PiT-2, which are encoded by *SLC20A1* and *SLC20A2* genes, respectively, in humans (35). They are expressed in a broad range of tissues with different amounts, and PiT-2 is suggested to have a role in renal Pi reabsorption. Inactivating mutations in *SLC20A2* are responsible for familial idiopathic basal ganglia calcification (IBGC), a disease characterized by vascular deposits of calcium/phosphate in the basal ganglia of the brain (36, 37). Thus, PiT-2 is likely to be involved in the maintenance of the physiological Pi level in cerebrospinal fluid. Recently, xenotropic and polytropic retrovirus receptor 1 (XPR1) has been shown to mediate Na^+^-independent Pi export from cells in mammals, and its inactivating mutations also cause IBGC (38). Regarding the type I Na^+^/Pi co-transporters, their physiological role has been shown to be the transport of organic anions rather than the Pi transport (39–41).

### Clinical Symptoms of Hyperphosphatemia and Hypophosphatemia

Hyperphosphatemia is associated with reduced renal Pi excretion or an excessive phosphate load. It causes ectopic calcification, which may lead to organ failure, gastroenteral bleeding, skin itchiness, keratitis, and tumoral calcinosis (42).

Hypophosphatemia is caused by insufficient intestinal Pi absorption, renal Pi wasting, or shift of Pi into cells (43). Chronic hypophosphatemia is often associated with renal Pi wasting diseases and leads to rickets/osteomalacia (43). Acute hypophosphatemia is associated with respiratory alkalosis, refeeding, diabetic ketoacidosis, malnutrition, and alcoholism (43). Since hypophosphatemia can be caused by transcellular shift of Pi into cells, low serum Pi levels do not always reflect the insufficient storage of Pi within cells. Extra-skeletal symptoms of hypophosphatemia include muscle dysfunction, arrhythmia, low cardiac contractility, hemolysis, white blood cell dysfunction, platelet dysfunction, myopathy, seizures, and fatigue (43). Systemic and extra-skeletal symptoms of hypophosphatemia are not so common as those of hypocalcemia, probably because Pi stored in the skeleton and the cells may prevent acute, severe hypophosphatemia. Hypocalcemia causes an increase in the permeability of plasma membrane of muscle and nerve cells to sodium ions, leading to tetany, cramps, and seizures. Since the intracellular level of calcium is much lower than its extracellular level, intracellular calcium signaling is easily influenced by an alteration in extracellular calcium level. Therefore, serum calcium levels should be more strictly controlled than serum Pi levels (44).

## FGF23-related Hyperphosphatemic and Hypophosphatemic Disorders

### Hyperphosphatemic Familial Tumoral Calcinosis

Since the FGF23/FGFR/αKlotho signaling is central in maintaining phosphate homeostasis, a disrupted or excessive signaling of this pathway will cause diseases with abnormal phosphate metabolism. Inactivating mutations in *FGF23, KLOTHO*, and *GALNT3* encoding GalNAc-T3, an enzyme required for *O*-glycosylation of FGF23, are responsible for hyperphosphatemic familial tumoral calcinosis (HFTC) associated with hyperphosphatemia, normal to elevated serum 1,25(OH)_2_D levels, and massive ectopic calcification (34, 45, 46).

### Autosomal Dominant Hypophosphatemic Rickets

An excessive action of FGF23 results in hypophosphatemic diseases with increased renal Pi wasting, an inappropriately low levels of serum 1,25(OH)_2_D and impaired skeletal mineralization (47). Autosomal dominant hypophosphatemic rickets (ADHR) is caused by missense mutations in the *FGF23* gene at Arg^176^ or Arg^179^, which make the protein resistant to inactivation by cleavage (48). Iron deficiency triggers the accumulation of uncleaved FGF23 in ADHR patients, leading to the manifestation of the hypophosphatemia and rickets/osteomalacia (49).

### X-Linked Hypophosphatemic Rickets and Autosomal Recessive Hypophosphatemic Rickets

Hypophosphatemic rickets/osteomalacia related to an excessive action of FGF23 is also caused by inactivating mutations in the *phosphate-regulating gene with homologies to endopeptidases, on the X chromosome (PHEX), dentin matrix protein 1 (DMP1), ectonucleotide pyrophosphatase/phosphodiesterase 1 (ENPP1)*, and *family with sequence similarity 20 C (FAM20C)* genes (48, 50–55). *PHEX* is responsible for X-linked hypophosphatemic rickets (XLH), the most common form of hereditary hypophosphatemic rickets (56). Although PHEX protein is suggested to function as a zinc-dependent protease based on its structure, its physiological substrates remain to be identified. DMP1, which is responsible for autosomal recessive hypophosphatemic rickets 1 (ARHR1), is an extracellular matrix protein belonging to the SIBLING (small integrin-binding ligand, N-linked glycoproteins) family. *ENPP1* encodes an enzyme which produces pyrophosphates (PPi) and is responsible for ARHR2. FAM20C is a kinase that phosphorylates various secreted proteins which include FGF23 and the SIBLING family such as DMP1. Inactivating mutations of FAM20C have been identified in patients with FGF23-related hypophosphatemia and dental abnormalities (54). Interestingly, PHEX, DMP1, and FAM20C are highly expressed in the osteocytes as well as FGF23 (57), suggesting that these molecules function as local negative regulators of FGF23 production and that osteocytes play a key role in phosphate homeostasis.

### Enhanced FGFR Signaling Might be Involved in FGF23 Overproduction in XLH and ARHR1

The mechanisms underlying the FGF23 overproduction are still largely unknown in most of the FGF23-related hypophosphatemic disorders. However, recent studies have suggested that an enhanced FGFR signaling might be a pathogenesis for the overproduction of FGF23 in osteocytes of XLH. In *Phex*-deficient *Hyp* mice (a murine model of human XLH), the osteocytic expressions of *Fgf1, Fgf2, Fgfr1–3*, and *Egr-1*, which is a target of activated FGFR signaling, were markedly increased compared to in wild-type mice, as well as that of *Fgf23* (57). In addition, the conditional deletion of *Fgfr1* in osteocytes partially restored the FGF23 overproduction and rescued the hypophosphatemia and mineralization defect in *Hyp* mice (58). These findings indicate the possible involvement of activated FGFR signaling in the FGF23 overproduction in *Hyp* osteocytes. Overproduction of FGF23 in *Dmp1*-knockout mice, a model for ARHR1, has also been attributed to enhancement of FGFR signaling in the bones (59). There is yet no human data on FGFR signaling in the osteoblasts/osteocytes of XLH and ARHR patients. However, a human disease called osteoglophonic dysplasia caused by activating mutations in FGFR1 is often associated with hypophosphatemia due to increased FGF23 levels (60), which also suggests the regulation of FGF23 production by FGFR signaling.

## Role of Phosphate in the Skeleton

### Matrix-Vesicle Mediated Mineralization and Pi

**Figure 1:**
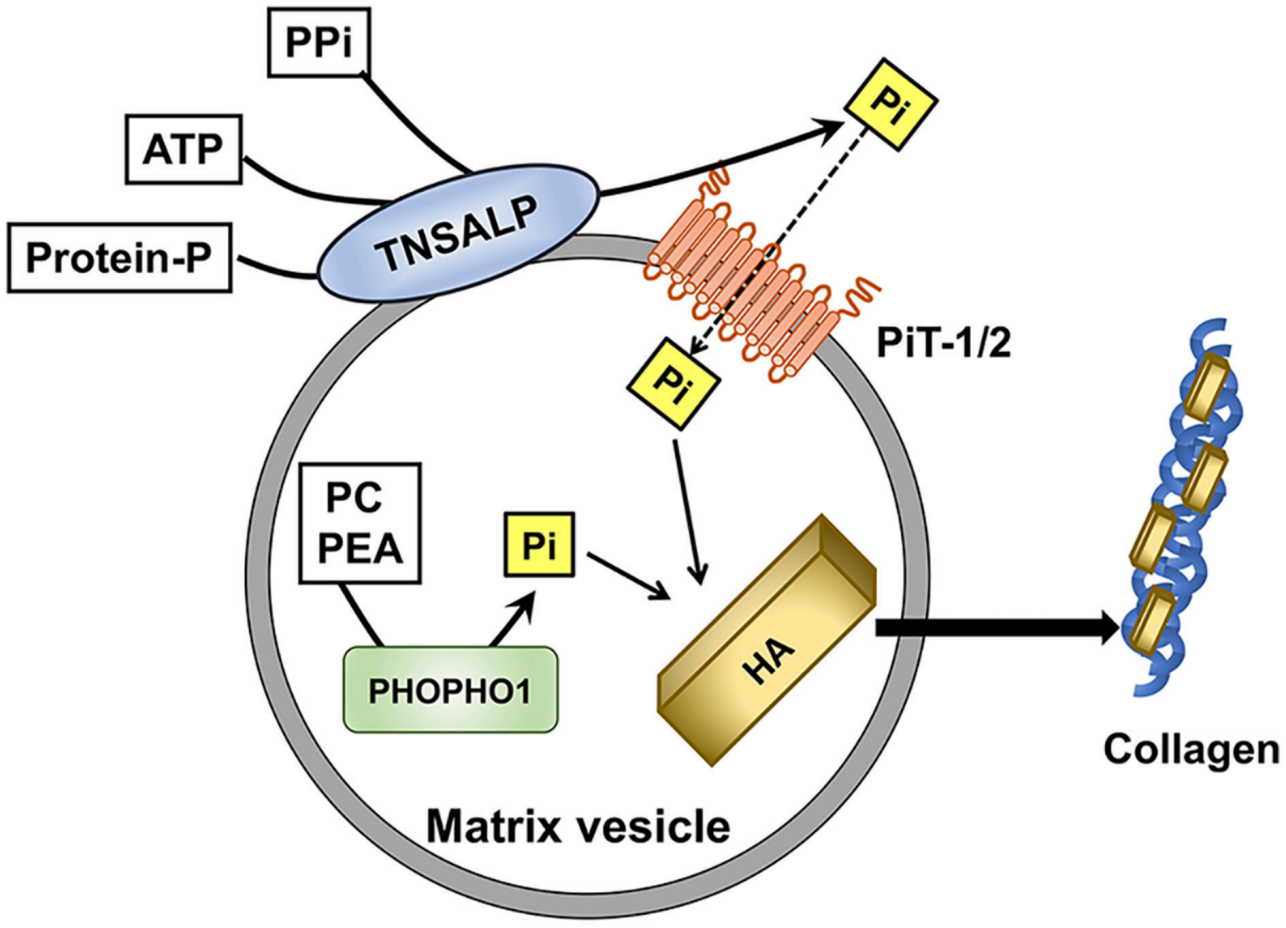
Role of Pi in the initiation of skeletal mineralization in matrix vesicles (MVs). Tissue-non-specific alkaline phosphatase (TNSALP) on the outer membrane of MVs hydrolyzes pyrophosphate(PPi), adenosine triphosphate (ATP), and protein-bound phosphate to produce Pi. Type III Na^+^/Pi co-transporters PiT-1 and PiT-2 mediate the Pi uptake into MVs. PHOSPHO1 produces Pi from phosphocholine (PC) and phosphoethanolamine (PEA) within MVs. Pi contributes to the formation of hydroxyapatite (HA), which will be deposited on the collagen fibrils in the extravesicular matrix.

Although the underlying mechanism for skeletal mineralization is not fully understood yet, involvement of matrix vesicles (MVs) has been suggested. MVs are the extracellular, small membranous structures produced by budding from the plasma membrane of osteoblasts and chondrocytes (61). They serve as the initial site of mineralization by rapidly taking up calcium and Pi ions to form hydroxyapatite crystals. The hydroxyapatite formed in MVs will then propagate on the collagen fibrils to mineralize the extracellular matrix (61, 62). MVs possess high activity of tissue-non-specific alkaline phosphatase (TNSALP), which functions as an ectoenzyme on the outer surface of the vesicles to hydrolyze PPi, adenosine triphosphate (ATP), and the protein-bound form of phosphate to generate orthophosphates (61, 63). PPi acts as an inhibitor against the formation of hydroxyapatite, and TNSALP facilitates the mineralization through the hydrolysis of PPi and the production of Pi. Inactivating mutations in TNSALP cause hypophosphatasia characterized by impaired skeletal mineralization (64). Another phosphatase called PHOSPHO1 has been identified to initiate mineralization by producing Pi from phosphocholine and phosphoethanolamine within MVs (65) (Figure 1).

Pi is transported into MVs by both Na^+^-dependent and Na^+^-independent components (66). The Na^+^-dependent Pi uptake into MVs appeared to be mediated by PiT-1 and PiT-2, similarly to the Pi uptake by the cells from which the MVs budded (67). Skeletal mineralization was normal in mice with hypomorphic expression of PiT-1 probably because a compensatory increase in the PiT-2 expression accounted for the sufficient Pi uptake (68). Thus, the net influx of Pi into MVs rather than the expression of each transporter seems to be more critical in the mineralization. With regard to the uptake of calcium ions into MVs, annexins which are calcium-binding proteins have been suggested to be involved (69).

Sufficient Pi supply is critical in MV-mediated mineralization. In rickets/osteomalacia, TNSALP is up-regulated compensatorily to supply the needs of Pi. In chondrocytic cells in culture, treatment with high Pi suppressed the expression of TNSALP within 24 h, indicating its responsiveness to Pi availability (9).

### Roles of Pi in Chondrocyte Apoptosis

In addition to its profound role in MV-mediated mineralization which occurs extracellularly, Pi also exerts its effects directly on the skeletal cells. In terminally differentiated chondrocytes, an elevated Pi level induces apoptosis (4–8), a process critical in endochondral ossification. Hypophosphatemia caused reduced apoptosis of hypertrophic chondrocytes and led to rickets in the XLH model mouse (*Hyp*) (70). Reduced apoptosis of hypertrophic chondrocytes was also reported in vitamin D receptor (VDR) knockout mice (71), and hypophosphatemia has been suggested to be a common etiologic factor among all types of rickets (72).

### Role of Extracellular Pi as a Regulator of Gene Expression in the Skeleton

Extracellular Pi also regulates gene expression. In 2000, Beck et al. demonstrated that extracellular Pi induced the expression of *osteopontin* (*Opn*) gene using a murine osteoblastic cell line MC3T3-E1 (12). Since then, a number of molecules have been identified to be responsive to the alteration in extracellular Pi levels. The Pi-responsive genes identified in osteoblasts include a cell-cycle related gene *cyclin D1* (73), and *Dmp1* (11). Since *Dmp1* is highly expressed in osteocytes, its up-regulation by an elevated extracellular Pi may facilitate the differentiation of osteoblastic cells into osteocytes. Extracellular Pi appears to regulate PPi metabolism as well, since *Enpp1* encoding a PPi-generating enzyme and *Ank* encoding a PPi transporter were also up-regulated by an elevated extracellular Pi (74).

The effects of extracellular Pi on the FGF23 expression in osteoblast-lineage cells has also been extensively investigated, but the results seem inconsistent both *in vivo* and *in vitro* (57, 75–79). In a recent human study, plasma FGF23 levels were transiently elevated 4 weeks after high phosphorus intake but returned to the baseline after 8 weeks (80). Treatment of cultured osteoblasts or osteocytes with elevated extracellular Pi increased the FGF23 expression only in a context-dependent manner (57, 78, 79, 81). Considering that an elevation of extracellular Pi induces the expression of *Dmp1* (11), Pi may increase FGF23 expression rather indirectly through facilitating the osteoblastic differentiation into osteocytes.

During endochondral skeletogenesis, phosphate content in the extracellular milieu of chondrocytes gradually increases (82). The effects of Pi on chondrocytes differ among the differentiation stages. In early chondrocytes, an increased extracellular Pi induced cyclin D1 expression to facilitate proliferation, and down-regulated *Alpl* encoding TNSALP (9). In more matured chondrocytes, high extracellular Pi up-regulated *Col10a1* encoding type X collagen (83) which is a marker for hypertrophic chondrocytes, and *matrix Gla protein* (*Mgp1*) (84) whose product inhibits mineralization.

## Signal Transduction Evoked by an Increased Extracellular Pi

### Increased Extracellular Pi Induces the Activation of Raf/MEK/ERK Pathway

Accumulating evidence has suggested the involvement of Raf/MEK (mitogen-activated protein kinase/ERK kinase)/ERK (extracellular signal-regulated kinase) pathway in the gene regulation by extracellular Pi. An increased extracellular Pi rapidly activated Raf/MEK/ERK pathway but did not other pathways such as p38MAPK pathway or JNK pathway (13). The activation of Raf/MEK/ERK pathway mediated the Pi-induced regulation of various genes including *Opn* (13), *Dmp1* (11), and *Mgp* (85) in osteoblastic MC3T3-E1 cells and *Mgp* (84), *Cyclin D1* and *Alpl* (9) in chondrocytic ATDC5 cells. Pi-induced activation of Raf/MEK/ERK pathway is also shared in the cells derived from extraskeletal tissues such as HEK293 cells (10).

### Increased Extracellular Pi Induces the Activation of FGFR

**Figure 2:**
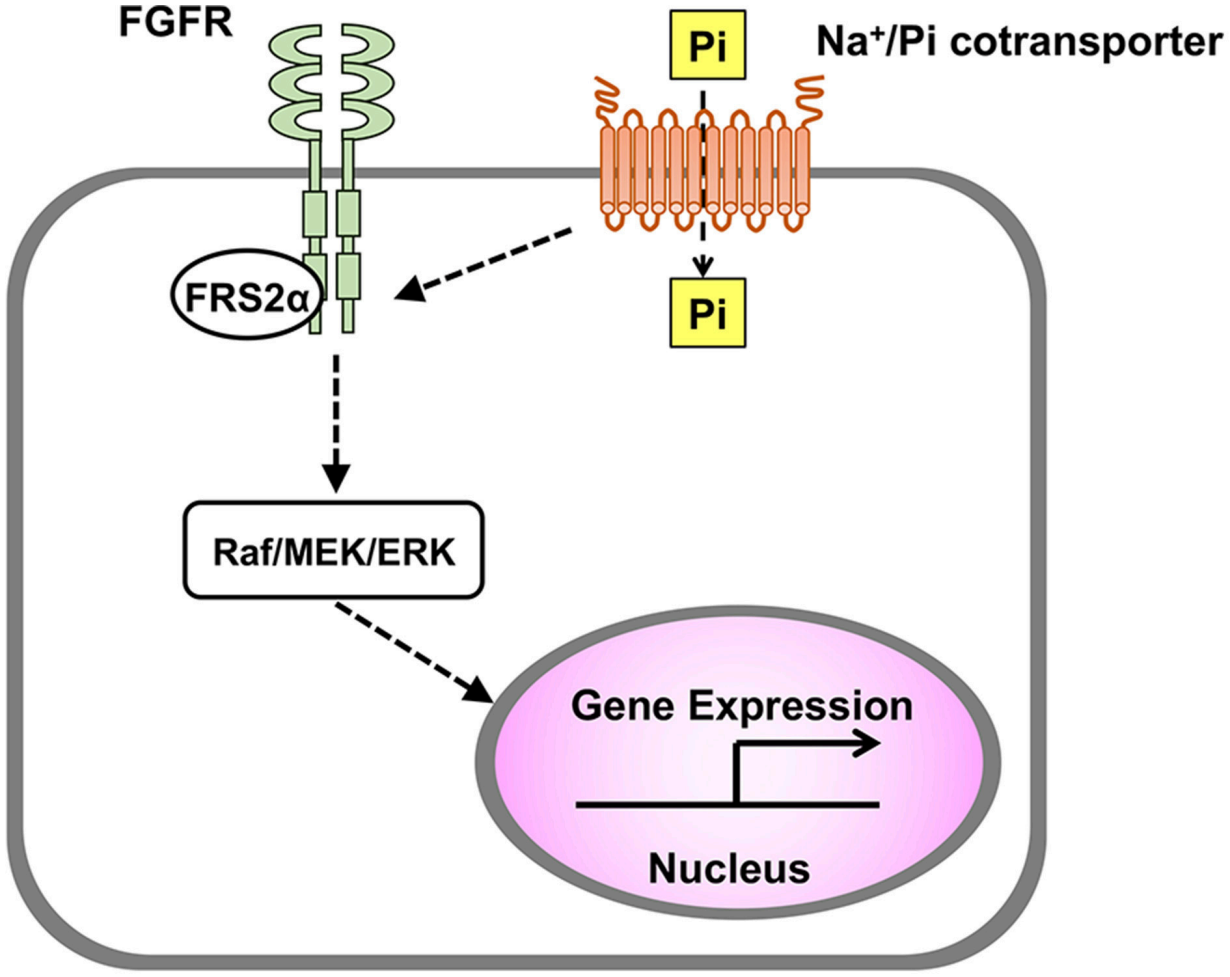
Transduction of signal evoked by extracellular Pi. In various cell types including osteoblasts and chondrocytes, an increased extracellular Pi induces the activation of Raf/MEK/ERK pathway to regulate gene expression, and this process is mediated by Na^+^/Pi cotransporter and FGFR.

Interestingly, an increased extracellular Pi also induces the activation of FGFR, which is one of the upstream signaling pathways of Raf/MEK/ERK cascade. In MC3T3-E1 cells, both an FGFR inhibitor and an MEK inhibitor abolished the up-regulation of Dmp1 by an increased extracellular Pi (11). In HEK293 cells, knockdown of FGFR1 diminished the phosphorylation of ERK1/2 induced by an increased extracellular Pi (10). These results suggest that FGFR plays a critical role in the transduction of the signaling evoked by an increased extracellular Pi. Knockdown experiments have also implicated that PiT-1 might mediate the Pi-induced signal transduction upstream of FGFR (Figure 2).

### A Possible Relationship Between FGFR and Pi-Sensing

Detection of Pi availability and adaptation are critical to maintain phosphate homeostasis. In unicellular organisms such as bacteria and yeast, the molecular mechanisms for Pi sensing and adaptation are well-defined (1). They use some types of Pi transporters and other molecules such as kinases to sense Pi levels. Although mammalian Pi sensors have not been identified yet, the responsiveness of mammalian cells to an alteration in extracellular Pi suggests that they also might sense and adapt to the Pi availability in their microenvironment. Considering the involvement of FGFR in the transduction of Pi-induced signal and the enhanced FGFR signaling in the osteoblasts/osteocytes in *Phex*-deficient *Hyp* mice (57, 58), impaired Pi-sensing in these cells might underlie the overproduction of FGF23 in XLH. Further study is required to clarify the molecular mechanisms by which mammals sense the Pi availability.

## Conclusion

Phosphate plays multiple functions in the skeleton. Outside of cells, it plays a pivotal role in the MV-mediated mineralization as a constituent of hydroxyapatite. Moreover, Pi induces apoptosis of hypertrophic chondrocytes. Extracellular Pi also triggers signals within the cell to regulate gene expression, although the role of intracellular Pi remains unclear. Hypophosphatemia causes rickets by reducing hydroxyapatite formation, impairing apoptosis of hypertrophic chondrocytes, and probably altering gense expression in the skeletal cells. *In vitro* studies have demonstrated the involvement of Na^+^/Pi cotransporter, FGFR, and Raf/MEK/ERK pathway in the transduction of Pi-evoked signal, and abnormalities in Pi-sensing might be the pathogenesis of some hypophosphatemic diseases such as XLH. Clarification of the mechanisms for Pi-sensing in human will contribute to the development of better strategies to treat the conditions with abnormal phosphate metabolism.

## Author Contributions

This manuscript was prepared by a joint effort of TM and KO. TM coordinated the process of manuscript preparation.

### Conflict of Interest Statement

The authors declare that the research was conducted in the absence of any commercial or financial relationships that could be construed as a potential conflict of interest.
